# iAstrocytes do not restrain T cell proliferation in vitro

**DOI:** 10.1186/s12868-023-00806-3

**Published:** 2023-06-07

**Authors:** Emanuela Colombo, Anthea De Angelis, Claudia Bassani, Francesca Ruffini, Linda Ottoboni, Livia Garzetti, Annamaria Finardi, Gianvito Martino, Roberto Furlan, Cinthia Farina

**Affiliations:** 1 Institute of Experimental Neurology (INSpe), Division of Neuroscience, IRCCS San Raffaele Hospital, Milan, Italy; 2 Vita-Salute San Raffaele University, Milan, Italy

**Keywords:** Astrocyte, iAstrocyte, Immunosuppression, Proliferation, T lymphocyte

## Abstract

The cross-talk between T cells and astrocytes occurring under physiological and, even more, neuroinflammatory conditions may profoundly impact the generation of adaptive immune responses in the nervous tissue. In this study, we used a standardized in vitro co-culture assay to investigate the immunomodulatory properties of astrocytes differing for age, sex, and species. Mouse neonatal astrocytes enhanced T cell vitality but suppressed T lymphocyte proliferation in response to mitogenic stimuli or myelin antigens, regardless of the Th1, Th2 or Th17 T cell phenotype. Studies comparing glia cells from adult and neonatal animals showed that adult astrocytes were more efficient in inhibiting T lymphocyte activation than neonatal astrocytes, regardless of their sex. Differently from primary cultures, mouse and human astrocytes derived from reprogrammed fibroblasts did not interfere with T cell proliferation. Overall, we describe a standardized astrocyte-T cell interaction in vitro assay and demonstrate that primary astrocytes and iAstrocytes may differ in modulating T cell function.

## Introduction

Central nervous system (CNS) immunosurveillance is a physiological process taken over by distinct immune cell types, including T lymphocytes [1]. In addition to being critical for detection and elimination of infectious agents and damage, central immunity sustains normal brain function, as e.g. lymphocyte deficient mice display impairment in spatial learning, memory [2, 3] and adult neurogenesis [4]. Under physiological conditions immune cells circulate in the cerebral spinal fluid, interstitial fluid, choroid plexus, meninges and perivascular spaces [1] and their migration into the CNS parenchyma is tightly regulated by a unique composition of barriers which, however, are breached in some neuroinflammatory disorders, including Multiple Sclerosis (MS). Studies in appropriate animal models for MS indicate that the generation of an adaptive immune response within the CNS may start with peripheral activation and differentiation of inflammatory myelin-reactive T cells that migrate into the CNS, exert a local immune response against self-antigens, thus triggering focal lesions of primary demyelination, glial proliferation and neurodegeneration [5]. Being part of the blood brain barrier, astrocytes may represent an early interaction partner for T cells and support or inhibit immune cell responses in the CNS [6, 7]. Astrocytes may act as antigen-presenting cells, as they may process and present CNS autoantigens to proinflammatory T cells [8–10]. Moreover, they may express adhesion and costimulatory molecules for T lymphocytes, thus sustaining the activation of differentiated T cells [6, 11]. On the other hand, astrocytes may also induce T cell anergy and apoptosis, and attenuate T cell proliferation [6, 8, 12]. Age and sexual dimorphism may affect glia cell phenotype and function [13–16], raising the issue whether the result of astrocyte-T cell interaction may change according to age and sex. Further, differences may exist between mouse and human astrocytes as demonstrated in a comparative ex vivo study [17], but the human counterpart is not readily accessible for in vitro studies. To overcome this hurdle, cell-reprogramming approaches have been developed to generate astrocytes from adult fibroblasts [18–21], which may model the phenotype of reactive astrocytes in MS lesions, as displaying e.g. high expression of cytokine and sphingosine receptors [22–24].

In this pilot study, we performed in vitro co-culture assays to investigate the effects on T lymphocyte proliferation exerted by astrocytes differing for age, sex, and species.

## Methods

### Animals

All procedures involving animals were authorized by the Institutional Animal Care and Use committee of the San Raffaele Scientific Institute and the Italian General Direction for Animal Health at the Ministry of Health. All methods were carried out in accordance with relevant ARRIVE guidelines. Pups were sacrificed by decapitation and adult animals by CO_2_ inhalation. Wild type C57BL6 mice were purchased from Harlan laboratories. Mice bearing a transgenic T cell receptor specific for myelin oligodendrocyte glycoprotein MOG_35–55_ peptide (2D2 mice) were provided by Furlan’s lab. All mice were bred in the institutional facility providing constant temperature (22  ± 1 °C), humidity (50%) and 12 h light/dark cycle. They had ad libitum access to food and water.

### Mouse primary astrocyte cultures

Neonatal astrocytes were prepared from brains of newborn C57BL6 wild type mice according to published protocols [25, 26]. Briefly, primary mixed glial cultures were established from the forebrains of 1 day-old male and/or female pups, mechanically dissociated and digested with 0.25% trypsin solution. Mixed cultures were maintained in complete astrocyte medium (DMEM-high glucose medium containing 1% antibiotics (Pen/Strep solution), 200 mM l-Glutamine, 100 mM Sodium Pyruvate and 10% FCS (all from Euroclone, Italy)). Ten day-old primary cultures were vigorously shaken to discard microglia and oligodendrocytes. The remaining adherent cells were detached and, following an adhesion step, non adherent cells were reseeded on poly-d-lysine (Sigma) coated flasks. Adult astrocyte cultures were established from 2 months-old male and/or female C57BL6 wild type mice. Brains were extracted and dissociated by combining enzymatic and mechanical dissociation using Adult Brain Dissociation Kit and the gentleMACS™ Dissociator (both from Miltenyi Biotec). After dissociation, myelin, cell debris and erythrocytes were removed and remaining cells were seeded on poly-d-lysine coated flasks in complete medium. Purity of astrocyte cultures was > 95% according to GFAP immunofluorescence.

### Astrocyte generation from mouse and human iPSC-NPC

Mouse and human neural precursor cells from induced pluripotent stem cells (iPSC-NPC), generated as described in [18, 23, 24], were provided by Martino’s lab. For the generation of iAstrocytes, mouse and human iPSC-NPCs were seeded in Geltrex coated flasks for 24 h. The day after, medium was changed to DMEM supplemented with 1% antibiotics, 200 mM l-Glutamine, 100 mM Sodium Pyruvate, 10% FCS and 0.3% N2 as described in [18, 23, 24]. Astrocytes were allowed to differentiate and checked for morphology and expression of astrocyte markers (GFAP, nestin, S100β and vimentin).

### Mouse spleen cell culture, CD4 + T cell isolation and differentiation

Spleens were extracted from adult C57BL6 wild type mice and mechanically dissociated as described in [26, 27]. After red cell lysis in ammonium chloride potassium lysis buffer (Sigma-Aldrich), spleen cells were resuspended in complete T cell medium (RPMI medium supplemented with 1% Pen/Strep solution, 200 mM l-Glutamine, 100 mM Sodium Pyruvate and 5% FCS). CD4 + T lymphocytes were purified from spleens of 2D2 mice by magnetic sorting with mouse CD4 + beads according to manufacturer’s instructions (Miltenyi Biotec). For differentiation in distinct Th subsets, CD4 + T cells were maintained in complete T cell medium in 96-well round-bottom plates at 200.000 cells/well. Cells were stimulated with anti-CD3 (5 µg/ml) and anti-CD28 (5 µg/ml) antibodies (BD Biosciences) and left grown for 7 days in the presence of either IL-12 (10 ng/ml) and anti–IL-4 (5 µg/ml; Th1 differentiating conditions), IL-4 (10 ng/ml) and anti-IFN-γ (10 ng/ml; Th2 differentiating conditions) or TGF-β (3 ng/ml) and IL-6 (30 ng/ml; Th17 differentiating conditions).

### Dendritic cell preparation

Bone marrow derived dendritic cells (DC) were prepared from flushed femur and tibia of C57BL6 mice and propagated in vitro for 1 week in Iscove’s medium (Life Technologies) supplemented with 1% antibiotics (Pen/Strep solution), 200 mM l-Glutamine, 10% FCS and recombinant mouse GM-CSF and IL-4 (both 25 µg/ml, R&D System).

### Proliferation assays

Splenocytes were cultured at 20,0000 cells/well in 96-well round bottom plates in absence or presence of 2000 adherent astrocytes/well and maintained in complete T cell medium. Alternatively, naïve or in vitro differentiated CD4 + T cells were added to astrocytes in the presence of DC (80.,00 T cells/50,000 DCs/2000 astrocytes/well) and, 24 h later, stimulated with T cell media containing 2.5 µg/ml Concanavalin A or 50 µg/ml MOG_35−55_ peptide. After 72 h incubation, cultures were pulsed for 18 h with 0.5 mCi/well of [3H] thymidine and harvested. Thymidine incorporation was measured from quadruplicate cultures per condition on a β-counter (PerkinElmer) and data were reported as counts per minute (c.p.m.) or proliferation index [c.p.m. + antigen/c.p.m. media]. Eventually, spleen cells or CD4 + T cells were labelled with Carboxyfluorescein succinimidyl ester (CFSE, Life Technologies) following manufacturer’s instruction before being added to astrocytes. After 72 h incubation, cells were stained for T cell marker CD3 (Biolegend) and analyzed by flow cytometry. 7AAD was used to determine cell viability (Biolegend). Samples were acquired at BD FACSCanto II (BD Biosciences) and analysed by FlowJo software (Tree Star Inc). In each experiment 2 to 3 technical replicates were analyzed.

### ELISA

IFN-γ, IL-4 and IL-17 levels were measured in supernatants from Th1, Th2 and Th17 CD4 + cells following 7 days of differentiation, washing, and 24 h restimulation with ConA. Samples were analyzed with ELISA MAX™ Standard Set (Biolegend) according to the manufacturer’s protocol. Colorimetric read-out was analyzed by Epoch Micro-Volume Spectrophotometer System (Biotek). Experiments were performed in duplicates.

### Immunofluorescence experiments

Astrocytes were plated on 12 mm diameter coverslips (8000 cells/coverslip), fixed in 4% PFA, blocked in PBS + 1% BSA (Merck) + 5% FCS and stained with primary antibodies. When needed, 0,1% Saponin (Merck) in PBS was added to blocking solution. Then, cells were incubated with appropriate species-specific Alexa Fluor 488/594-conjugated secondary antibodies (Thermo Fisher Scientific), counterstained with 4′,6-diamidino-2-phenylindole (DAPI, Sigma) and mounted with fluorescent mounting medium (Agilent). The following primary antibodies were used: rabbit anti-GFAP (Agilent), mouse anti-nestin (Merck Millipore), mouse anti-vimentin (Abcam), rabbit anti-S100β (Abcam). The following secondary antibodies were used: Alexa Fluor 488 donkey anti-rabbit IgG (H + L), Alexa Fluor 594 donkey anti-rabbit IgG (H + L), Alexa Fluor 594 donkey anti-mouse IgG (H + L) (all from Thermo Fisher Scientific). Fluorescence images were captured at fluorescence microscope (Leica DM5500B). LASAF software was used for image acquisition and ImageJ (download at: http://rsbweb.nih.gov/ij/) software was used for image analysis.

### Statistical analyses

Data in figures are presented as mean ± standard deviation (SD) or standard error of the mean (SEM) as indicated in figure legends. The exact number of samples or independent experiments is reported in figure legends. Normality of the distribution was assessed by Kolmogorov-Smirnov statistics. Unpaired t-test was performed to compare means. All p-values were two-sided and subjected to a significance level of 0.05. In figures, asterisks denote statistical significance as *p  < 0.05; ** p  < 0.01; *** p  < 0.001. Statistical analyses were performed in Excel or GraphPad Prism.

## Results

### Interaction with primary mouse astrocytes inhibits T cell proliferation

**Fig. 1:**
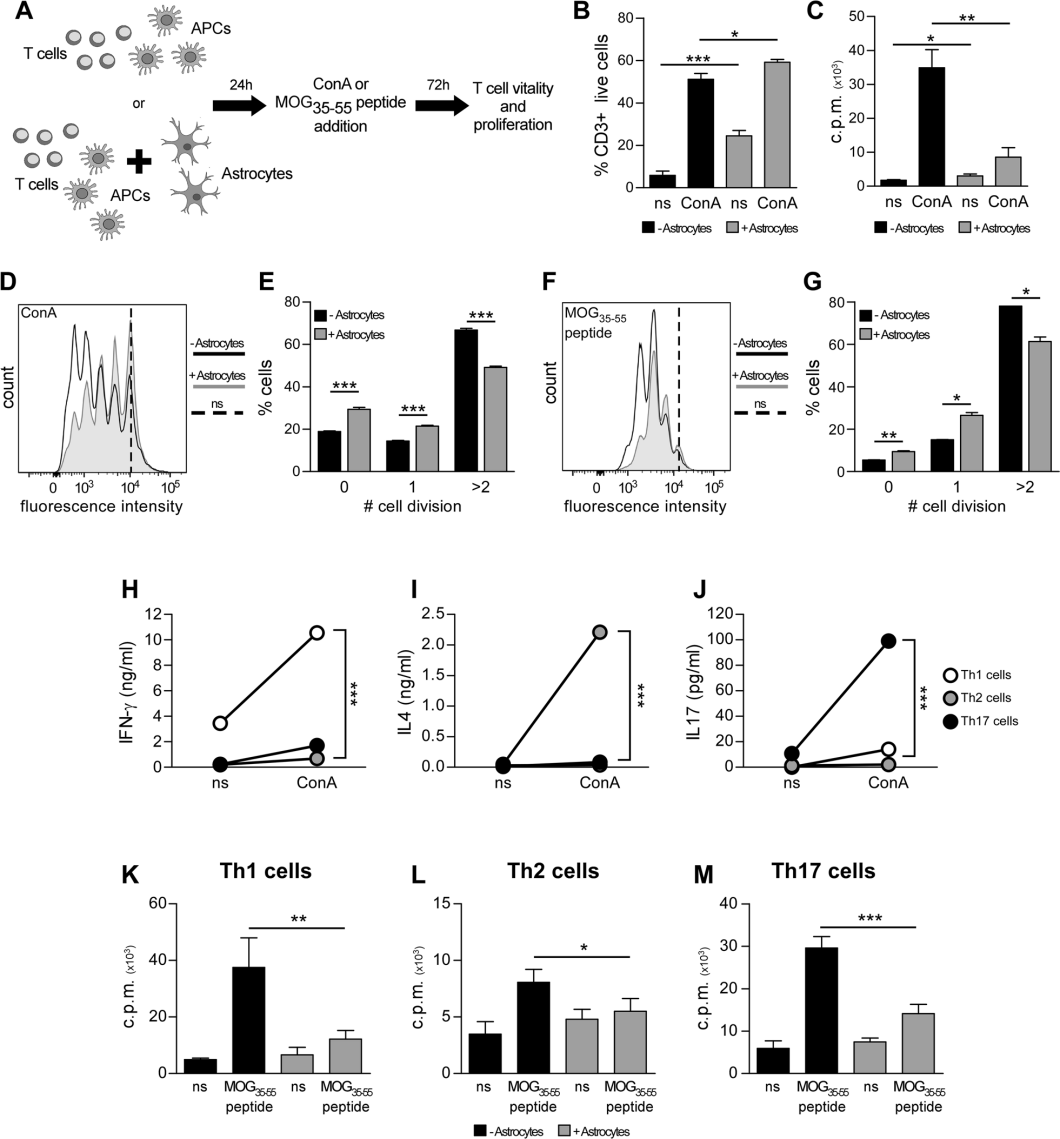
Astrocyte-T cell co-culture enhances immune cell vitality but inhibits ConA-mediated and antigen-specific T cell proliferation. **A** Experimental scheme: T cells were seeded with antigen presenting cells in absence or presence of astrocytes, and received media containing ConA or MOG_35 − 55_ peptide 24 h later. After 72 h incubation cells were harvested and T cell vitality and proliferation were addressed by flow cytometry and/or thymidine incorporation. **B** Percentage of living CD3 + T cells as assessed by 7AAD incorporation in unstimulated (ns) or ConA-supplemented cultures without (black bars) or with (grey bars) primary neonatal astrocytes. **C** ConA-induced T cell proliferation assessed by [3H] thymidine incorporation in cultures without (black bars) or with (grey bars) primary neonatal astrocytes. Data are reported as counts per minute (c.p.m.). **D** CFSE levels (x axis) in mouse CD3 + T cells in ConA-activated cultures without (black bars) or with (grey bars) neonatal astrocytes. **E** Percentage of T lymphocytes that underwent 0, 1 or > 2 cell cycle divisions. (**F**, **G**) CFSE levels in mouse 2D2 CD4 + T cells after exposure to MOG_35 −55_ peptide in cultures without (black bars) or with (grey bars) neonatal astrocytes (**F**), and relative quantification (**G**). In D and F dotted lines represent mean CFSE levels in unstimulated cells (ns). **H**–**J** TNF-α (**H**), IL-4 (**I**) and IL-17 (**J**) released by 2D2 CD4 + Th1 (white dots), Th2 (grey dots) and Th17 (black dots) under resting conditions or after 24 h ConA stimulation. **K**–**M** MOG_35 −55_ peptide induced proliferation of 2D2 CD4 + Th1 (**K**), Th2 (**L**), or Th17 (**M**) lymphocytes cultured without (black bars) or with (grey bars) neonatal astrocytes. Representative data of one out of 2–5 independent experiments are shown. In (**B**–**C**, **E**, **G**, **H**–**M**) data are reported as mean ± SD. Statistical analysis was performed using T test. *p-value < 0.05, **p-value < 0.01, ***p-value < 0.001

We addressed astrocyte potential in modulating T cell proliferation by establishing an ad hoc astrocyte-splenocyte co-culture assay (Fig. 1A). Primary mouse astrocytes from newborn C57BL6 mice were seeded and allowed to achieve complete adherence. Freshly prepared spleen cells from adult C57BL6 mice were cultured alone in complete T cell medium or added to neonatal astrocytes in an astrocyte: splenocyte ratio equal to 1:100. After 24 h, cultures were stimulated with the T cell mitogen Concanavalin A (ConA) or the MOG_35−55_ peptide for 72 h, and then pulsed with [3H] thymidine to measure T cell proliferation (Fig. 1A). Alternatively, splenocytes were labelled with carboxyfluorescein diacetate succinimidyl ester (CFSE), added to astrocytes, and stained for CD3 marker after 72 h stimulation. Finally, T cell vitality and proliferation were assessed by 7-AAD and CFSE dilution respectively at the flow cytometer. Most of the unstimulated T cells died after 3 days in culture, while ConA treatment increased the percentage of live cells (Fig. 1B, black columns). Importantly, exposure to astrocytes promoted in vitro immune cell survival, especially when T cells were in resting conditions (Fig. 1B, grey bars). However, while ConA-induced T cell proliferation measured by [3H] thymidine incorporation was massive in control cultures, it was greatly inhibited by astrocytes (Fig. 1C). Similar results were obtained with the CFSE-based proliferation assay (Fig. 1D). In fact, in the absence of astrocytes about 33% of the T cells had performed no more than one cycle of cell division, and 66% had proliferated two or more times (Fig. 1E, black columns). On the contrary, when T lymphocytes were exposed to astrocytes, about 51% of the cells had not proliferated or had had one cycle of cell division and only 49% of the cells had proliferated two or more times (Fig. 1E, grey columns). To test whether astrocytes could modulate myelin-specific T cell responses, we purified CD4 + T cells from splenocytes of transgenic 2D2 mice. In these animals, all CD4 + T lymphocytes express a transgenic myelin oligodendrocyte glycoprotein (MOG)-specific T-cell receptor and are thus responsive to stimulation with MOG_35−55_ peptide when presented by dendritic cells. Purified CD4 + 2D2 T cells were stained with CFSE, co-cultured with dendritic cells in the absence or presence of astrocytes and stimulated after 24 h with MOG_35−55_ peptide. Cytofluorimetric analysis revealed that astrocytes blocked T lymphocyte proliferation in response to the specific MOG_35−55_ antigen (Fig. 1F, G).

To verify whether distinct T cell phenotypes were susceptible to astrocyte-mediated suppression, we skewed 2D2 CD4 T cell phenotype under Th1, Th2 or Th17 polarizing conditions and repeated our coculture assays. Appropriate T cell polarization was confirmed by Elisa for lineage-specific cytokines (IFN-γ, IL-4 and IL-17 for Th1, Th2 and Th17 respectively) in supernatants of Con-A activated T cells (Fig. 1H–J). Co-culture experiments exposing Th1, Th2 or Th17 MOG-specific 2D2 T cells to neonatal astrocytes showed that astrocyte-mediated immunosuppression was effective regardless of T cell phenotype (Fig. 1K–M).

**Fig. 2:**
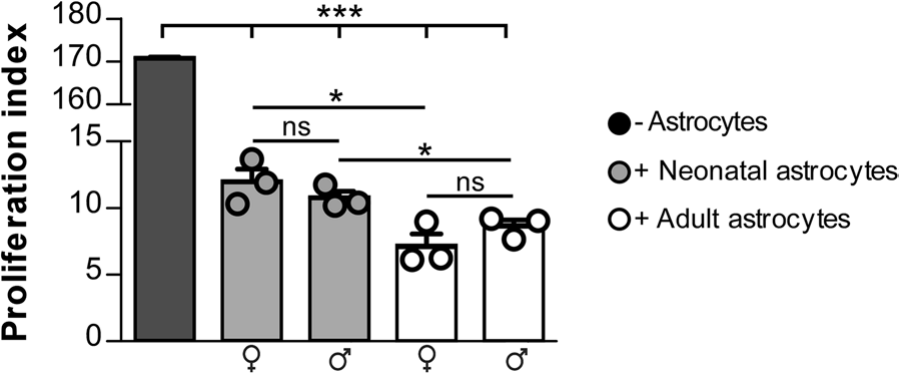
Adult astrocytes display stronger immunosuppression than neonatal cells, regardless of sex differences. ConA-induced T cell proliferation in cultures without (black bars) astrocytes, with female and male neonatal astrocytes (grey bars) or female and male adult astrocytes (white bars). Each dot represents a distinct astrocyte preparation. Representative data of one out of 2 independent experiments are reported as proliferation index [c.p.m. + antigen/c.p.m. media] and represented as mean ± SEM

Finally, we investigated whether age or sex could affect the immunosuppressive property of astrocytes. The astrocyte-splenocyte co-culture assay was repeated with distinct preparations of primary astrocytes isolated from female or male adult mice and compared in parallel assays with female or male neonatal astrocytes. While female and male glia cells were equally potent in blocking T cell proliferation (Fig. 2), adult astrocytes were significantly more effective than neonatal cells (Fig. 2 grey and white bars).

### iPSC-NPC derived astrocytes do not exert the same immune-regulatory properties as primary astrocytes

**Fig. 3:**
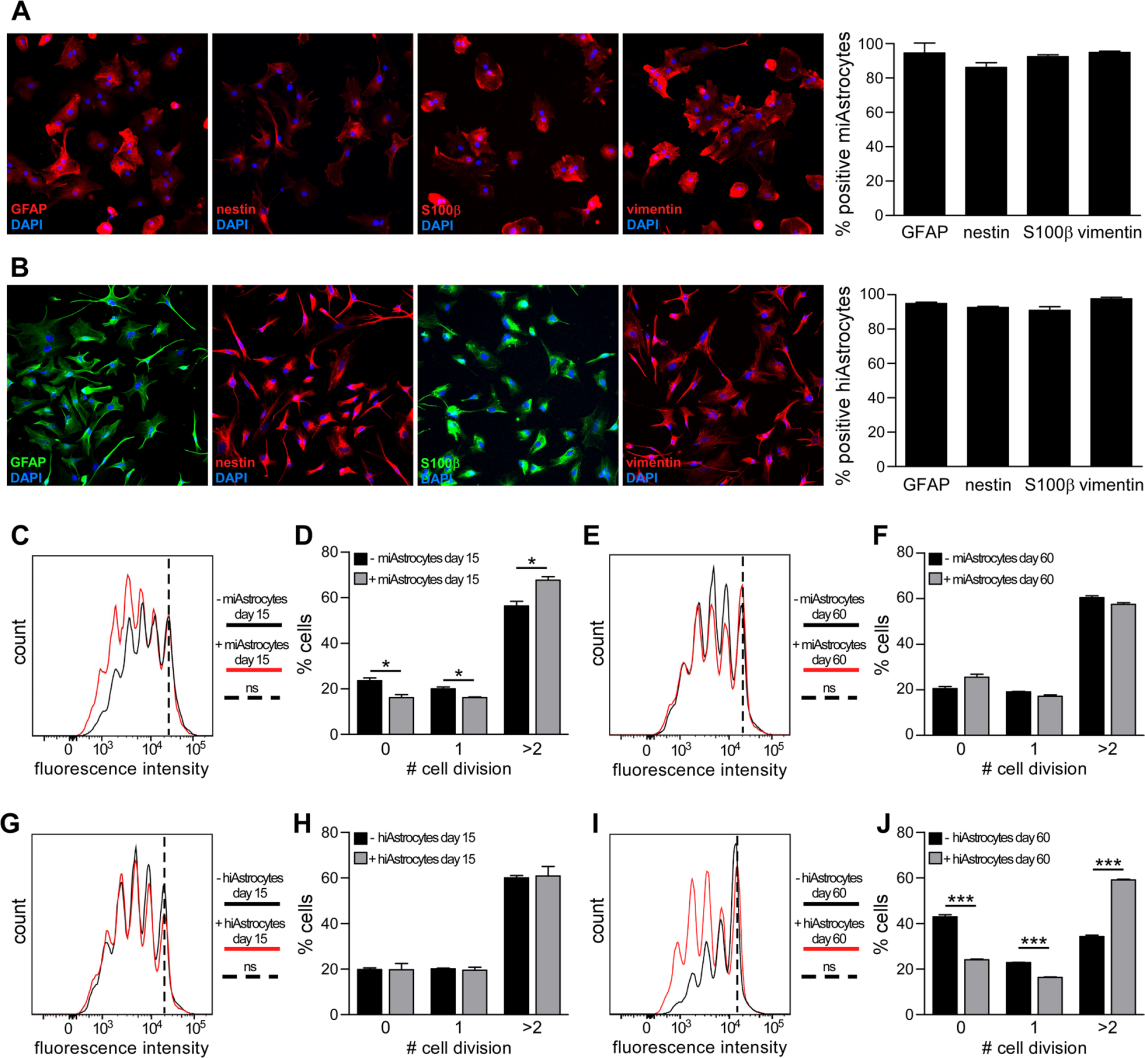
iPSC-NPC derived astrocytes do not hinder T cell proliferation. **A**, **B** Fluorescence images showing representative stainings for GFAP, nestin, S100β and vimentin in mouse (**A**) or human (**B**) iAstrocytes and percentage of cells positive for astrocyte markers (right panels). DAPI was used for nuclear staining. (**C**–**F**) CFSE levels in mouse ConA-activated CD3 + T lymphocytes cultured without (black lines) or with (red line) mouse iAstrocytes differentiated for 15 days (**C**–**D**) or 60 days (**E**–**F**). Graphs in D and F report the percentage of T cells which underwent 0, 1 or > 2 cell divisions. (**G**–**J**) CFSE levels in mouse ConA-activated CD3 + T lymphocytes cultured without (black lines) or with (red line) human iAstrocytes differentiated for 15 days (**G**, **H**) or 60 days (**I**–**J**). In **C**, **E**, **G** and **I** dotted lines represent mean CFSE levels in unstimulated cells (ns). In **C**–**J** representative data of one out of 3–7 independent experiments are shown and reported as mean ± SD. In A and B graphs report data from two iAstrocyte cell lines and bars represent SEM. Statistical analysis was performed using T test. *p-value < 0.05, ***p-value < 0.001

Mouse and human iAstrocytes were differentiated from iPSC-NPCs as previously described [18, 23, 24]. iAstrocytes were cultured and sampled at different time points to assess their phenotype. Immunofluorescence experiments confirmed that mouse and human iAstrocytes displayed typical astrocyte morphology and expressed astrocyte markers (GFAP, nestin, S100β and vimentin) already after 2 weeks in culture (not shown) and maintained this phenotype at day 60 (Fig. 3A, B) after differentiation induction. We then tested iAstrocytes, differentiated for 15 or 60 days, in the astrocyte-splenocyte co-culture assays and assessed CFSE-based T cell proliferation by flow cytometry. As shown in Fig. 3, both mouse and human iAstrocytes were either unable to interfere with T cell proliferation or even supported it (miAstrocytes day 15, Fig. 3C–F; hiAstrocytes day 60, Figure G–J). Thus, differently from primary cultures, mouse and human iAstrocytes did not suppress ConA-induced T cell proliferation at any tested time points (Fig. 3).

## Discussion

In this study, we described a standardized astrocyte-T cell co-culture assay that allowed appropriate comparison of distinct factors such as astrocyte sex, age and source, or T cell phenotype. Further, we translated our findings from animal to human cells.

Neonatal astrocytes were efficient in attenuating T lymphocyte proliferation induced by either mitogenic or antigenic stimuli and this effect was not due to reduction in T cell number because of lymphocyte death. In fact, we reported that exposure to a few astrocytes (astrocyte: T cell ratio 1:40) boosted the vitality of both resting and activated T cells. On the other hand, in vitro studies using different astrocyte-T cell ratios up to 2:1 indicate that a major presence of astrocytes may induce T cell apoptosis via different mechanisms (e.g. release of Galectin 9 and FasL [8, 28–31]). Overall, these observations suggest that local enrichment in glial or T cells may dictate T cell fate and function.

Astrocyte-mediated immunosuppression was equally efficient towards myelin-reactive Th1, Th17 and Th2 CD4 + T cells (our study and [12]). Published data indicate that astrocytes may also support survival and activation of regulatory T cells [32, 33]. In our experimental setup we cannot exclude that astrocytes can regulate T cell proliferation via an action on antigen presenting cells (APC), however, data from the literature indicate that exposure to astrocytes alone can inhibit T-cell proliferation in vitro in absence of APCs [12, 34, 35], thus demonstrating a direct possible effect of astrocytes on T cells. Data from the literature indicate that cell contact mediated interactions between astrocytes and T cells may inhibit T lymphocyte proliferation [8] and foster the acquisition of an immunosuppressive phenotype by T cells via upregulation of CD39 and CD73 ectonucleotidases on lymphocyte surface [34]. On the other hand, astrocytes may attenuate T cell activation via the cytotoxic T lymphocyte antigen (CTLA)-4 even when contacts between astrocytes and T cells are prevented by cell culture inserts [12], suggesting that also soluble factors may mediate astrocyte-dependent immunosuppression. Accordingly, inhibition of T cell proliferation can be achieved after exposure to mouse or human astrocyte-conditioned media [35–37]. Astrocytes are highly secretory cells and may produce pro-inflammatory as well as anti-inflammatory cytokines [38, 39], and factors sustaining T-cell recruitment as well as anergy [34, 40, 41]. Most information on astrocyte immune competence detailed above has been obtained from in vitro studies on rodent cells from neonatal animals. However, astrocytes undergo phenotypical and functional changes during postnatal maturation [13, 15]. Gene expression studies describe changes in the transcriptional repertoires of astrocytes isolated from newborn or adult mice [13, 42, 43]. Further, the comparison of primary astrocytes from neonatal or adult animals revealed differences in cellular electrophysiology [44] and regulation of neural stem cell proliferation [45]. Here we demonstrated that cells isolated from adult animals were more efficient in suppressing T cell proliferation compared to neonatal astrocytes, suggesting that astrocytes may better dampen T cell function in the adult tissue. Our data extends to glial immune competence the concept of maturation-related changes in astrocyte function and highlights the necessity to include age as a biological variable in research on astroglia cells.

Sex may also affect morphology, phenotype and function of glia cells. A recent RNA sequencing study revealed distinct patterns of gene expression in male versus female astrocytes across development and from birth to the adult stage [13], suggesting that astroglia mature faster in male mice than in female mice. Importantly, sex impacts also on glial expression of inflammatory genes in response to inflammatory challenges or oxidative stress [14, 16, 46]. Male and female astrocytes may show divergent responses in animal models of neuroinflammation [47], and in vitro after exposure to pathological insults, such as LPS [46], ethanol [48] or oxygen deprivation [49]. These dissimilar responses may reflect sex-specific pathological vulnerabilities or rescue strategies to restore tissue homeostasis. Our study did not find any difference in immunosuppressive competence exerted by astrocytes from age-matched male and female mice, indicating that this specific property of astroglia cells is independent of sex.

Astrocytes are crucial regulators of tissue response to neuroinflammation [50] and are emerging as useful candidates for pre-clinic transplantation studies in neuroinflammatory disorders [51]. Due to the need for reliable and sustainable sources of astrocytes, the goal of many research laboratories has become the efficient derivation of functional rodent and human iAstrocytes [18–21, 23, 24]. iAstrocytes exhibit in vitro biological features similar to primary cells as they may take up and transport neurotransmitters [20, 21, 23, 24], generate calcium flux [20, 21, 52] and display phagocytic activity [52]. iAstrocytes hold the potential to respond to inflammatory signals and promote tissue damage. In fact, similarly to primary cells, iAstrocytes express receptors for inflammatory cytokines [24, 26] and respond to inflammatory mediators by NFkB translocation [23, 24] and production of cytokines [20, 23, 52] and neurotoxic mediators [20, 23, 24]. However, our study revealed an important functional distinction between rodent primary cells and iAstrocytes in the modulation of T cell activation, as iAstrocytes did not exhibit any immunosuppressive activity in our co-culture assays. The same observation was confirmed for human iAstrocytes as well.

In conclusion, though primary astrocytes and iAstrocytes may display similar innate immune competence in terms of primary generation of inflammatory responses, they may differ in terms of support of T cell function in the context of inflammation, a peculiarity that must be taken into account when planning in vitro and in vivo studies with iAstrocytes.

## Data Availability

The data sets used and/or analyzed during the current study are available from the corresponding author.

## Abbreviations

APC: Antigen presenting cell
CFSE: Carboxyfluorescein succinimidyl ester
CNS: Central nervous system
ConA: Concanavalin A
DC: Dendritic cells
iPSC: Induced pluripotent stem cells
NPCs: Neural precursor cells
MOG: Myelin oligodendrocyte glycoprotein
MS: Multiple sclerosis
